# Phylogenetic and Phylodynamic Analyses of HCV Strains Circulating among Patients Using Injectable Drugs in Central Italy

**DOI:** 10.3390/microorganisms9071432

**Published:** 2021-07-02

**Authors:** Claudia Minosse, Leonidas Salichos, Chiara Taibi, Ilaria Luzzitelli, Daniela Nardozi, Maria Rosaria Capobianchi, Gianpiero D’Offizi, Fiona McPhee, Anna Rosa Garbuglia

**Affiliations:** 1 Laboratory of Virology, “Lazzaro Spallanzani” National Institute for Infectious Diseases, IRCCS, 00149 Rome, Italy; claudia.minosse@inmi.it (C.M.); nardozi.daniela@gmail.com (D.N.); maria.capobianchi@inmi.it (M.R.C.); 2 Program in Computational Biology and Bioinformatics, Yale University, New Haven, CT 06520, USA; leonidas.salichos@yale.edu; 3 Biological and Chemical Sciences, New York Institute of Technology, New York, NY 11568, USA; 4 Hepatology and Infectious Diseases Unit, “Lazzaro Spallanzani” National Institute for Infectious Diseases, IRCCS, 00149 Rome, Italy; chiara.taibi@inmi.it (C.T.); ilaria.luzzitelli@inmi.it (I.L.); gianpiero.doffizi@inmi.it (G.D.); 5 Bristol-Myers Squibb Research and Development, Cambridge, MA 02142, USA; fiona.mcphee@bms.com

**Keywords:** hepatitis C virus (HCV), HCV genotypes, molecular epidemiology, phylogeny, people who use drugs (PWUD), viral evolution

## Abstract

Approximately 71 million people worldwide are infected with the hepatitis C virus (HCV). Injectable drug use represents the most common route of transmission in Europe and other developed countries. We studied the molecular characteristics of the HCV infection among mono-infected people who used drugs (PWUD) in Italy. Among 208 PWUD with anti-HCV antibodies, 101 (48.6%) were HCV RNA-positive, the majority (47%) were infected with the HCV genotype (Gt)1a, followed by Gt3a (34.9%), Gt4 (9.1%), Gt1b (4.5%), and Gt2 (4.5%). Bayesian phylogenetic analyses of clustered HCV NS5B sequences from 66 HCV-positive PWUDs with available plasma samples indicated age and neighborhood proximity as the most common characteristics between closely related HCV strains. Population dynamics, as measured by a coalescent Bayesian skyline analysis, revealed an increase in HCV Gt1a infections from the mid-1980s to mid-1990s. While HCV Gt3a infections were first detected in the 1980s, patient numbers with this genotype subtype remained relatively constant. For both Gt1a and Gt3a, Birth–Death Bayesian Skyline analyses produced higher reproduction numbers post 2014. For earlier time intervals, slow growths were observed for both Gt1a and Gt3a with reproduction numbers (Re) of approximately 1. The evolutionary rates for Gt1a and Gt3a were estimated as 2.23 × 10^−4^ and 3.85 × 10^−4^, respectively.

## 1. Introduction

The WHO estimated that approximately 71 million people worldwide are infected with the hepatitis C virus (HCV), with 1.75 million new infections being reported every year [1]. This virus is a blood-borne pathogen. Before the screening for anti-HCV antibodies was introduced in 1990, blood transfusion was the main risk factor for HCV infection [2]. In 1993, Western countries started using a nucleic acid amplification (NAT) test to detect the presence of HCV RNA. The availability of more sensitive tests led to a reduction in HCV prevalence. In Italy in 2013, the HCV RNA incidence rate in first-time blood donors was 2.5 per 100,000 [3]. Illicit drug users (IDUs) have been the predominant HCV-infected population since the early 2000s [4]. The WHO estimated that nearly 13 million individuals worldwide are IUDs, of which 67% are living with HCV [5]. Despite the route of transmission being similar for HCV and HIV, the transmission rates appear to vary. The HCV is estimated to be approximately ten times more transmissible than HIV, as only 30% of infected IDUs have HIV. Seroprevalence of HCV antibodies is very high among people who inject drugs (PWID); up to 90% of PWID have HCV antibodies compared with approximately 10% with HIV seroprevalence [6,7]. Preventive strategies, such as the reduction of syringe exchange with better aseptic technique compliance, have facilitated the reduction of HCV transmission by drug injection. However, numerous studies have shown that the sharing of drug paraphernalia is responsible for the spread of HCV among both IDUs and non-IDUs [8,9]. Specifically, the sharing of a sniffer device is a relevant factor in the spread of HCV among non-IDUs [10,11]. Changes in drug consumption and the awareness of safer drug injection practices have reduced the incidence of HCV transmission. In France, for instance, the cumulative incidence of HCV infection in IDUs was 40–50% over the last five years with the implementation of safer drug practices; this is compared with 90% in the early 1990s [12,13]. In Italy, in 2016, a national surveillance analysis at the Italian Centers for substance dependence indicated that approximately half a million of the population were people who use drugs (PWUD) and 30% tested positive for HCV [14]. The HCV status of these patients remains poorly understood, especially the variability in HCV transmission dynamics among this special population. As reported for other Western countries [15], genotypes 1a (Gt1a) and 3 (Gt3) are the most prevalent among PWID. HCV Gt4 prevalence is also increasing and drug use seems to be a key factor for the spread of this genotype [16], combined with people emigrating from African and Asian countries. To date, no phylogenetic or phylodynamic studies have been performed that focus on the correlation of drug use with the HCV genotype and the age of the patient.

The aims of this study were to: (1) establish the genotypic profile of HCV infection in Centers for Drug Addiction (SerD) located in the metropolitan area of Rome, Italy, (2) characterize the HCV strains in PWUD since genetic data on these strains are limited, and (3) analyze the potential variation in the viral population over the last two to three decades.

## 2. Materials and Methods

### 2.1. Population Study

To evaluate the distribution of the HCV genotype in PWUD, a cross-study analysis was conducted using data collected from two SerD outpatients’ services from the metropolitan area of Rome, Lazio. Between March 2018 and January 2020, 720 sera from individuals attending two SerDs, located in a suburban area of Rome, were tested for the presence of anti-HCV antibodies. The study design and procedures were previously described (Taibi et al., Manuscript submitted). All participants enrolled in the study provided written informed consent. Participants were interviewed face-to-face by trained healthcare professionals using a standardized questionnaire, which contained the following queries: sex, age, residency, type of drug use, behavioral information, HIV serostatus, and anti-HCV medication, if relevant. Datasets describing the demographics and the behavioral and clinical history for each participant were merged and shared with the Hepatology Unit Collaborative group in order to explore any associations with HCV sequence clustering. Variables used for the analysis included: age, gender, and the type of drug first injected and subsequent use over time. Age was used as a categorical variable, with age group (≤34; 35–44; ≥45) in the descriptive analysis. Data regarding the presumed date of primary HCV infection were collected retrospectively.

The study was approved by the Institutional Ethics Board (approval number code n 49/2017).

### 2.2. HCV Viral Load Determination and HCV Genotyping

All participants were initially tested for anti-HCV antibodies (Architect, Abbott). HCV-positive participants were recalled to the INMI L Spallanzani Hospital where plasma samples were collected for the HCV RNA viral load and the HCV genotyping evaluations. The HCV RNA viral load was measured using a commercial real-time RT-PCR assay (RealTime™ HCV, Abbott Molecular Inc., Des Plaines, IL, USA), as specified by the manufacturer. The lower limit of detection (LLOD) was 12 IU/mL. The HCV genotypes were assigned using the Abbott RealTime HCV Genotype II Assay (Abbott Molecular Inc., Des Plaines, IL, USA). All samples identified as Gt2, Gt3, Gt4, and Gt6 were subtyped by phylogenetic analysis.

### 2.3. HCV Molecular Characterization

The NS5B region was amplified from the HCV RNA-positive samples. Briefly, total nucleic acid extraction from the HCV RNA-positive plasma samples was achieved using a QIASYMPHONY automated instrument (QIAGEN, Hilden, Germany), and eluted in a buffer (60 µL) according to the manufacturer’s instructions. The NS5B region was amplified from the extracted viral RNA (5 µL) by nested RT-PCR employing the One-Step RT-PCR kit (Cat#210212, Qiagen, Hilden, Germany) and PCR TaqGold (TermoFisher Scientific, Waltham, MA, USA), using primers previously published by Pilon et al. [17]. Amplicons were then sequenced in both directions using a Big Dye Cycle Sequencing Standard Kit and the dideoxynucleotide chain terminator method in an ABI Prism 3500 system (Applied Biosystems, Foster City, CA, USA). All resultant nucleotide sequences were edited and aligned using the BioEdit program [18]. Sequences were aligned with CLUSTALW [19]. Furthermore, patient-derived HCV NS5B sequences from patients, either attending our outpatient service (marked with “Q”), or previously hospitalized at the INMI L Spallanzani Hospital (marked with “Pt”), were considered for phylogenetic analysis. Moreover, additional NS5B sequences from other countries were downloaded from the Los Alamos National Laboratory (LANL) HCV sequences database if the following criteria were met: (1) known country of origin, (2) known genotype and, (3) sequences already published in peer-reviewed journals. Phylogenetic trees were built using the maximum likelihood analysis and employing a general time-reversible (GRT) substitution model implemented using the Molecular Evolution Genetic Analysis (MEGA) software v. 10 [20]. Trees were visualized with FigTree (Rambaut A, v. 1.4.3-October 2016; http://tree.bio.ed.ac.uk/software/figtree/, accessed on 24 March 2021).

Sequences were associated with a particular cluster if the nucleotides differed by less than 3% and the bootstrap support value was greater than 70% [21]. Monophyletic clusters and phylogenetic trees were derived from sequences that clustered using the maximum likelihood method. Pairwise time comparisons were calculated by contrasting estimated dates of infection between case sequences that clustered versus those that did not. All nucleotide sequences derived in this study were uploaded to GenBank (accession numbers MW927596-MW927703).

The study cohort patient-derived HCV NS5B PCR fragments were analyzed for resistance-associated substitutions (RASs).

### 2.4. Phylodynamic Analysis

MAFFT [22] was employed to build multiple sequence alignments based on nucleotide data. BEAST v2.6.3 [23,24] was used to implement a Bayesian analysis with time constraints based on sampling dates. All analyses were performed using a general time-reversible (GTR) gamma site model with four categories and a strict molecular clock. A strict molecular clock was selected since our sequences were: (1) homochronous and contemporary, (2) representative of recent outbreaks, (3) limited in size, and (4) of interest in relation to the direct measurements of population effect size. Using Beast v2.6.3, the Markov chain Monte Carlo algorithm was run for 100 million states and sampled at every 10,000. Tree management and visualization were performed using Figtree v1.4.4 (Rambaut A, v. 1.4.4-November 2018; http://tree.bio.ed.ac.uk/software/figtree/, accessed on 25 March 2021).

#### 2.4.1. All Subtypes

For the global tree, a prior “Birth and Death model” was employed, allowing for the inclusion of date information. We selected our prior based on a coalescent Bayesian skyline (BSKY) model [25], which suggested a logistic growth with a sharp population increase during the first half of the 20th century, otherwise with constant effective population sizes. The final tree enabled the determination of HCV subtypes and the identification of groups of patients with a high HCV sequence similarity (see below). The algorithm convergence was confirmed using Tracer v1.7.1 (Rambaut A and Drummond AJ, v. 1.6-May 2014; http://tree.bio.ed.ac.uk/software/tracer/, accessed on 15 March 2021) through the evaluation of parameter traces and an effective sample size (ESS > 200). 

#### 2.4.2. Gt1a and Gt3a Outbreaks Using Global Heterochronous Sequences

The most prevalent genotype groups, represented by Gt1a and Gt3a, were examined separately using reference sequences with date information. For these groups, the population growth was estimated using a coalescent BSKY model that utilized sequence dates. BSKY implementation supported the use of a “Birth and Death model” as a prior in Gt1a and Gt3a outbreaks. Known and identified clusters from (I) enabled us to calibrate the individual subtype trees of heterochronous sequences and estimate their divergence times in order to calculate the earliest common ancestor for Italian SerDs and non-SerDs cohort sequences for each genotype (see III). The algorithm’s convergence was confirmed using Tracer v1.7.1 through evaluation of parameter traces and an effective sample size (ESS > 200). Effective temporal reproduction numbers (Re) and uninfectious rates δ were estimated using a “Birth and Death Skyline serial model”. To distinguish between global and temporal Re, the dimension parameter was set to 1 and 10, respectively, as our prior to calculate the reproductive number. Additionally, ‘Beta’ was replaced by rho prior to 999, to signal that our sequences were a subset of total HCV infections.

#### 2.4.3. Gt1a and Gt3a Outbreak in Italy Using Homochronous Sequences 

By removing any reference sequences and focusing on homochronous sequences from Italy, both “Birth and Death Skyline Contemporary” [26] and “Phylodynamics” were used. “Birth Death SIR (contemp)” models [27] were used to estimate temporal Re and uninfectious rates δ, accessed on 26 March 2021. To distinguish between global and temporal Re, the dimension parameter was set to 1 and 10, respectively, as our prior to calculating a reproductive number. Additionally, ‘Beta’ was replaced by rho prior to 999, to signal that our sequences were a subset of total HCV infections. The results were further analyzed and visualized using Tracer v1.7.1 and the library bdskytools (https://github.com/laduplessis/bdskytools) in R (http://www.r-project.org/, accessed on 29 March 2021). Date information regarding the age of the common ancestral sequence was used in R plots derived from (II) to calibrate the 10 dimensions. The algorithm’s convergence was assessed using Tracer v1.7.1 through the evaluation of parameter traces and an effective sample size (ESS). While global Re values converged for both models, the last two out of ten (most recent) temporal Re values only converged in one of the two models for Gt1a, indicative of a recent and rapid increase in the HCV reproduction number, which deviated from a “Birth and Death logistic growth”.

#### 2.4.4. Calibrated Tree Using Epidemiological Data and Identified Clusters

The study dataset contained sequences from previously identified interpersonal relationships. Additional clusters were also detected where sequences differed by 3% at the most, and the node support indicated at least a 95% posterior probability. Pairwise genetic similarity for all of the sequences in our dataset was calculated using MEGAX [20], software for the statistical analysis of molecular evolution.

### 2.5. Statistical Analysis

The HCV genotype distribution and the demographic variable were compared using the chi-squared test. A *p*-value < 0.05 was considered significant. All statistical analyses were performed using the Statistical Analysis System (SAS) (SAS Institute Inc., Cary, NC, USA).

## 3. Results

### 3.1. Demographics

A total of 215 patients were identified as being anti-HCV antibody-positive; 7 were coinfected with HIV and excluded from the study. Among 208 HCV mono-infected patients, 101 (48.6%) were HCV RNA-positive. The average age of all 101 patients was 51 ± 8.43 (mean ± SD) years old. The HCV genotype distribution did not differ by age or HCV RNA viral load (Table 1). A mixed infection (Gt3a + Gt4) was identified in 1 patient using the Abbott real-time method (noted as Gt3a in Table 1).

Plasma samples were available for 66 of the 101 HCV RNA positive patients. These 66 patients were treatment-naive to DAA therapy. Genotype analysis using the sequenced HCV NS5B region of these samples agreed with real-time Abbott results. For the one patient identified as harboring a mixed infection (Gt3a + Gt4) by using the Abbott real-time method, only a Gt3a sequence was detected by using Sanger sequencing. Sanger sequences representing Gt1, Gt2, Gt3, and Gt4 by using the Abbott platform were subtyped. Overall, no HCV mixed infections were detected. The distribution of the HCV genotypes in this subgroup reflected the prevalence observed for the main cohort (Table 1: Gt1a, 45.6%; Gt1b, 4.0%; Gt2, 4.0%; Gt3a, 35.6%; Gt4, 11.9%). The majority of the PWUD were infected with Gt1a (*n* = 31, 47%) and Gt3a (*n* = 23, 34.9%), followed by Gt4 (*n* = 6, 9.1%; *n* = 5 with Gt4d and *n* = 1 with undefined Gt4 subtype), Gt1b (*n* = 3, 4.5%), and Gt2 (*n* = 3, 4.5%) (Table 2). None of the known NS5B RASs were detected in any of these patients.

The majority of participants were male (54/66; 81.8%) and >45 years old (81.8%). The nucleotide diversity of Gt1a, Gt3a, Gt1b, Gt2b, and Gt4d strains ranged from 2.0–6.9% (mean 4.5%), 1.8–6.1% (mean 5.3%), 5.1–6.7% (mean 5.6%), 2.9–4.5% (mean 3.9%), and 3.2% to 8.2% (mean 4.5%), respectively. None of the participants who were first tested for HCV during the 2018–2019 period harbored Gt1b, underlining how its transmission is not strictly associated with drug use. Both Gt1a and Gt3a were predominant irrespective of the participant’s age or date tested result, while the Gt1b and Gt4 cases were registered before 2018. The respective prevalence of Gt1a and Gt3a over the following time intervals were: 45.2% and 33.3% from <1990 to 2001, 47.1% and 41.2% from 2002 to 2013, and 50% and 33.3% from 2014 to 2019.

### 3.2. Phylogenetic Analysis

A global HCV phylogenetic analysis was performed using a total of 329 sequences, including selected reference sequences (Tables S1–S4). This analysis was performed to identify specific subtype clusters with 66 sequences from SerD patients and 42 sequences (Gt1a, *n* = 9; Gt1b, *n* = 7; Gt2c, *n* = 9; Gt3a, *n* = 9; Gt3b, *n* = 1; Gt4d, *n* = 5; Gt6, *n* = 2) from non SerD Italian patients living in Italy.

**Figure 1:**
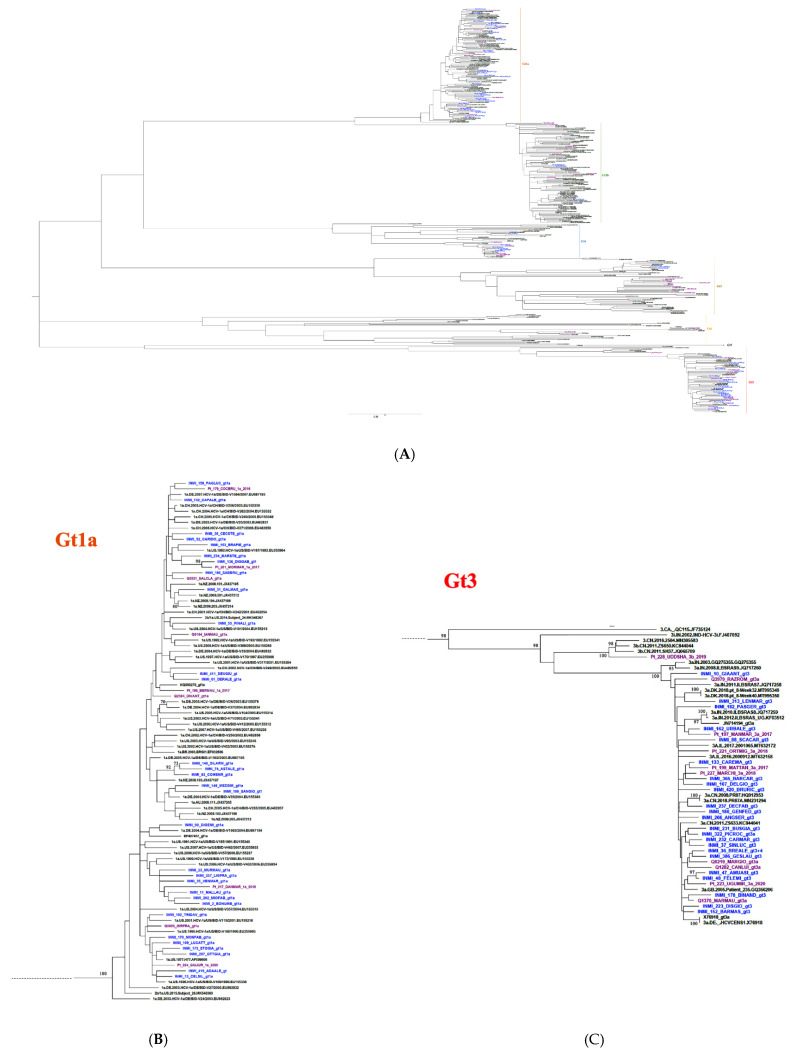
Maximum likelihood phylogenetic tree. Phylogenetic analysis showing (**A**) sequence clusters for SerD (in blue) and non-SerD (in purple) patients with HCV, (**B**) sequence cluster for SerD (in blue) and non-SerD (in purple) patients infected with HCV Gt1a, (**C**) sequence cluster for SerD (in blue) and non-SerD (in purple) patients infected with HCV Gt3a.

The phylogenetic tree, generated from HCV NS5B sequences (Figure 1), showed SerD (sequences indicated as INMI) that the patient-derived sequences were interdispersed. Four clusters were observed containing two or three INMI strains (INMI_140_SIALRM, INMI_74_ASTALE and INMI_83_COMENR in Gt1a; INMI_154_CAPALF and INMI_255_CUGROB in Gt4d; INMI_120_GILANZ, INMI_114_FUBSAN, and INMI_238_SGRENZ in Gt2c; INMI_47_AMUASI and INMI_48_ FELENI in Gt3a).

**Figure 2:**
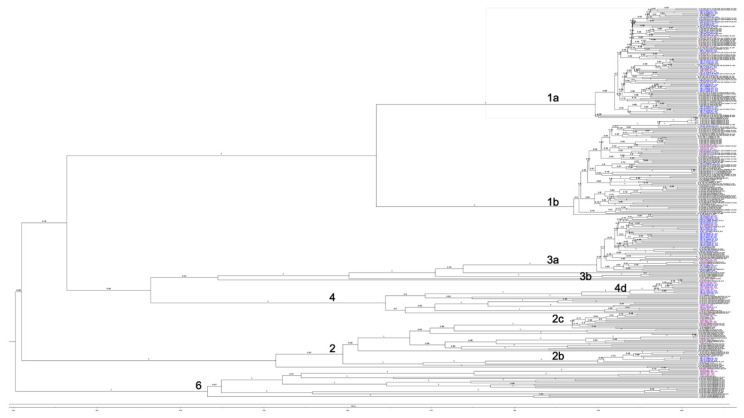
Genotyping and identification of sequence clusters for SerD (in blue) and non-SerD (in purple) HCV-infected patients. A Bayesian phylogenetic tree was generated assuming a prior Birth and Death population growth, using a GTR gamma site model with four inferred categories, to allow for gamma rate heterogeneity in our model.

The most common characteristics of patients with clustered sequences were age (INMI_83_COMENR, INMI_140_SILARM, and INMI_174_ASTALE) and the district where they lived. Comparing the HCV Gt3a sequences within one cluster, two strains were identified that included patients involved in a relationship (INMI_47_AMUASI and INMI_48_FELENI) and patients living in the same neighborhood/district (INMI_223_DISGIO and INMI_152_BARMAS). Of the three patients infected with HCV Gt2, subtyping of their NS5B sequences indicated alignment with Gt2b in the same cluster (Figure 2). These patients were friends and usually took drugs intravenously together sharing syringes. Only four study participants snorted their drugs; two were infected with HCV Gt1a (INMI_159_PAGLUC_gt1a; INMI_52_CARIDO_gt1a), one with Gt3a (INMI_133_CAREMA_gt3), and one with Gt4 (INMI_212_CIPRIT_gt4). Their sequences did not cluster with other SerD patients (Figure 1).

The earliest divergent patient, based on our data, was INMI_174_ASTALE, who reported becoming addicted to drugs in 1995 and was diagnosed with HCV in 2017. Therefore, the time of infection (earliest divergent node for this group) was estimated as being between 1995 and 2000. By 2000, both INMI_83_COMENR and INMI_140_SILARM had tested positive with HCV. For the Gt2b cluster, patients INMI_114_FUBSAN, INMI_238_SGRENZ, and INMI_120_GILENZ were acquaintances. For the Gt3a cluster, patients INMI_47_AMUASI and INMI_48_FELENI were involved in a personal relationship. While INMI_48_FELENI admitted to becoming addicted in 1993 and was confirmed as being HCV-positive in 1995, and the respective dates for INMI_47_AMUASI were 2012 and 2017. We, therefore, estimated the date of their common ancestral HCV sequence as being, approximately, 2012. Our criteria-based analysis highlighted three additional clusters (Table 3). Based on the respective time constraints, a tree calibration was implemented using Figtree by setting an offset of 2020 and a scale factor of 0.1 on inferred trees using a Birth and Death prior, as suggested by a coalescent Bayesian skyline analysis. 

**Figure 3:**
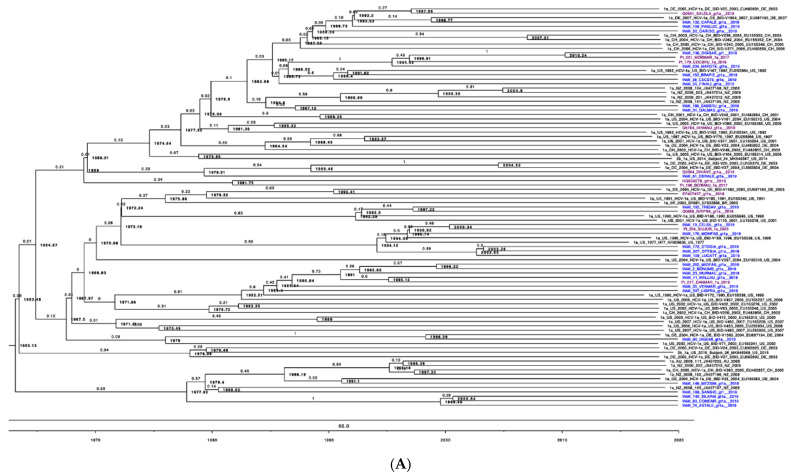

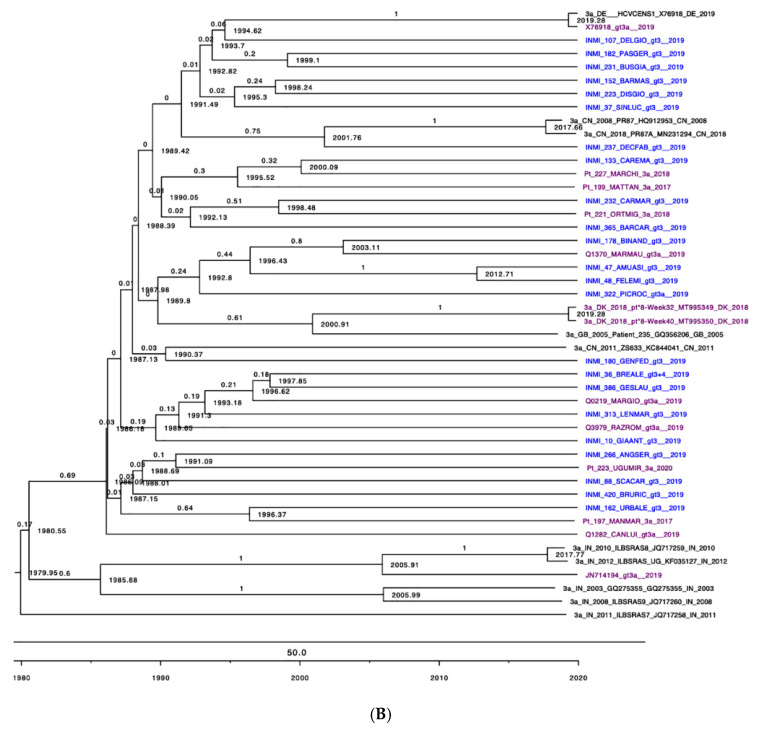
Bayesian phylogenetic tree with a “Birth and Death” population growth prior, using a GTR gamma site model with four categories and a strict molecular clock. Node values represent the year of divergence, while branch values depict the posterior probability. Tree calibration was implemented based on epidemiological data and known clusters. Using Figtree, an offset of 2020 and a scale factor of 0.1 were applied. (**A**) Time of divergence is dated for SerD (in blue) and non-SerD (in purple) patients with HCV Gt1a. (**B**) Time of divergence is dated for SerD (in blue) and non-SerD (in purple) patients with HCV Gt3a.

Using both sequence similarity and internode posterior probability, we were able to identify nine sequence clusters with a high affinity and sequence similarity (Figure 3 and Table 3). 

INMI_11_MALLAU_gt1a and INMI_23_MURMAU_gt1a were two 60-year-old patients, who started using heroin in 1978 and were confirmed as being HCV-positive in 1990. Although they both attended the same SerD, neither admitted that they knew of each other while being interviewed. INMI_109_LUCATT_gt1a_2019 (62 years old) and INMI_173_STOGIA_gt1a_2019 (54 years old) lived in the same district, attended the same SerD, and were acquainted; the former tested positive for HCV in 1986 while the latter tested positive in 1990.

For study participants infected with the most prevalent HCV genotypes, Gt1a and Gt3a, the focus was to estimate when these genotype sequences were first detected in Italy and to subsequently evaluate their population dynamics. Epidemiological and sequence information from known data were used to calibrate the tree (see methods). For Gt1a, results suggested this genotype was first detected in Italy around the early 1960s (1963). At the same time, a coalescent Bayesian skyline analysis indicated an increase in the HCV Gt1a population from the mid-1980s to mid-1990s. The analysis of the Gt3a sequences indicated that this genotype was first detected in the 1980s, however, the overall Gt3a population has remained relatively constant from the 1980′s onwards. The evolutionary rates for Gt1a and Gt3a were estimated as 2.23 × 10^−4^ and 3.85 × 10^−4^, respectively. 

**Figure 4:**
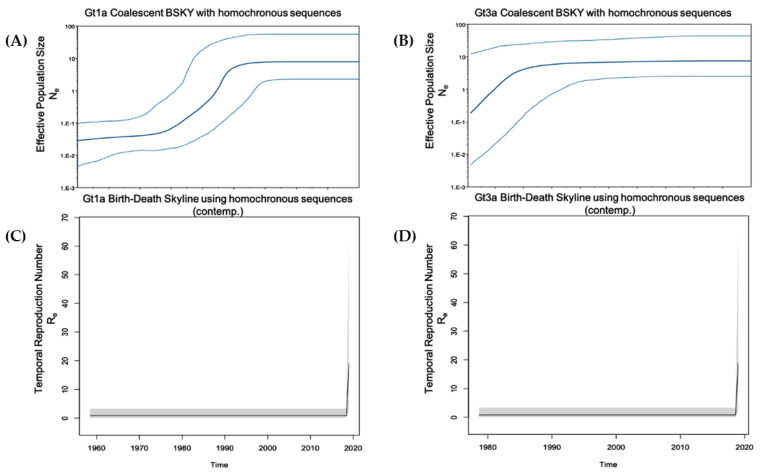
Population growth and temporal reproduction numbers for Gt1a and Gt3a using homochronous (SerD and non-SerD) reference sequences. (**A**,**B**) The Coalescent Bayesian skyline model combined with a GTR gamma site model (4 categories) and a strict molecular clock with sequence date constraints were used to estimate effective population size. The solid middle line represents the median, while the upper and lower lines represent the 95% high posterior density interval estimate of Ne over time. (**C**,**D**) The Birth-Death Skyline (contemporary) model was used to estimate temporal reproduction numbers from homochronous sequences. Figure **A**/**C** and **B**/**D** are aligned on the same time scale (x-axis).

To examine the population dynamics of the most predominant genotypes (Gt1a and Gt3a) in PWUD in Italy, a Birth and Death Bayesian Skyline contemporary analysis was performed using homochronous sequences from SerD and non-SerD cohorts, applying two modifications (see methods). Convergence of our models was observed for most dimensions except for the last two. These two dimensions represented the most recent time intervals and demonstrated a very sharp increase in reproduction numbers (Figure 4, Table S5), which may have impacted data convergence when using the Birth and Death model.

**Figure 5:**
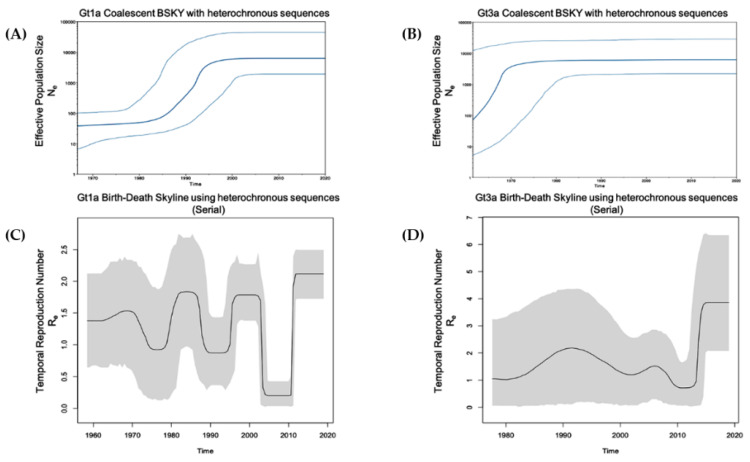
Population growth and temporal reproduction numbers for Gt1a and Gt3a using only (SerD and non-SerD) heterochronous reference sequences. (**A**,**B**) The Coalescent Bayesian skyline model combined with a GTR gamma site model (4 categories) and a strict molecular clock with sequence date constraints were used to estimate effective population size. The solid middle line represents the median, while the upper and lower lines represent the 95% high posterior density interval estimate of Ne over time. (**C**,**D**) The Birth-Death Skyline (serial) model was used to estimate temporal reproduction numbers from heterochronous sequences.

For Gt1a, resultant reproduction numbers were 2.8 and 25, for the two most recent time intervals, respectively, which were calculated from a fully converged analysis. For Gt3a, although convergence of all ten dimensions was not possible, resultant reproduction numbers were similarly high for the last two dimensions, which were the 9th (7.3 and 8.7) and 10th (18.7 and 19.2) dimensions. For the earlier time intervals evaluated, there appeared to be a slow growth in both Gt1a and Gt3a since their initial entry into Italy (Re ~1) (Figure 5, Table S6).

Given the extremely high Re calculated for the Italian sequences analysed after 2015, a Birth and Death Bayesian Skyline serial analysis was performed that included our heterochronous reference sequences. Even though this analysis expanded the viral population beyond the SerD and non-SerD Italian sequences, an increased effective Re was established for Gt1a and Gt3a during the study period, confirming a very recent spike.

## 4. Discussion

In this study, HCV infection among PWUD was evaluated around the metropolitan area of Rome. HCV RNA was detected in 48.6% of participants who tested positive for anti-HCV antibodies. This prevalence was considerably higher when compared with reports for the general population, although slightly less than reported in 2009 by Stroffolini et al. where 68.3% of HCV-positive IDUs were also viremic [16]. We could not associate HCV RNA prevalence with specific age groups since 82% of the study participants were >45 years old. Gt1b was infrequently observed (prevalence, 4.5%) in comparison to the general population [28], suggesting a different route of transmission. The prevalence of Gt4 (9.1%) was in line with previously described results over the past few decades in Italy and other Mediterranean basin countries [29,30,31]. In fact, the prevalence of this genotype in Europe ranges from 8.2% to 20% [32,33]. In France, the prevalence of Gt4 has increased from 4% in 1990 to greater than 11% over the last decade [34,35]. In Greece, Gt4 accounts for approximately 15% of all HCV infections [36,37]. Furthermore, our results showed a reduced prevalence of Gt4 when compared with other certain populations, such as men who have sex with men [38].

Gt1a was the most prevalent genotype (47%) in all evaluated age categories, followed by Gt3a (34.9%). In line with our results, Stroffolini et al. reported that the most common HCV genotypes among intravenous IDUs with Gt1a and Gt3a prevalence of 47.9% and 39.7%, respectively [16]. The marked differences between Gt1a and Gt1b strengthen the hypothesis that these two subtypes have different transmission routes. Gt1a was first introduced into Italy in the early 1970s, and coalescent Bayesian skyline analysis highlighted an exponential increase in Gt1a incidence between the 1980s and 1990s (Figure 4). This finding is in accordance with the younger age of infected HCV Gt1a patients, especially when compared with Gt1b, the main genotype detected in older chronically infected HCV patients [15]. Thus, there appears to be a dispersal origin. In the majority of cases, PWUD strains are intermixed with non-PWUD strains suggesting that Gt1a became a widely distributed subtype, affecting different patient demographics. The Gt1a sequences formed distinct clusters, distributed throughout the Bayesian phylogenetic tree without any distinct pattern. This is likely the result of a widespread and long-term distribution of Gt1a within the global population, and it is consistent with observations such as those in Greece regarding Gt1a dispersal among IDUs [39].

A higher prevalence of Gt1a was observed across all of the evaluated time intervals when compared to Gt3a (Table 2). Gt1a prevalence was 45.2% from <1990 to 2001 and its prevalence has gradually increased through subsequent years (47.1% from 2002 to 2013 and 50% from 2014 to 2019). Gt3a accounted for 33.3% of HCV infections from <1990 to 2001, increased to 41.2% from 2002 to 2013 and decreased to 33.3% from 2014 to 2019. Gt1a prevalence was always greater than 40% for each evaluated time period, which may be related to patient age. Approximately 80% of these patients were over 45 years old and 87.9% began using drugs before 1995. Of these patients, 23 were anti-HCV antibody-positive and 12 (39.7%) were infected with Gt1a, as shown in the phylodynamic analysis, demonstrating an increase of Gt1a infection between the mid-1980s and mid-1990s. Nevertheless, since the date of the first HCV positivity is estimated, it is possible that the primary infection occurred during a time period significantly prior to the first serological test in the early years of drug use, as reported in the literature [40]. Conversely, Gt3a prevalence ranged from 33–41% across all evaluated time intervals, in accordance with results from the coalescent Bayesian analysis illustrated in the skyline plot, which underlined a constant increase of Gt3a from 1986 onwards. The epidemiology of increased Gt3a transmission among the PWUD population is unknown. It is possible that this genotype, which is endemic in India, was introduced into Italy through PWUD who visited India in the 1970s when opium was easily accessible [41,42]. Moreover, the increased spread of Gt3a may have been related to the migration of people, including PWUD, from Eastern Europe after the Berlin wall came down in 1989 [43]. The lower prevalence of Gt3a at all of the analysed time intervals may be due to the reduced transmission of this genotype in comparison with Gt1a or, conversely, a higher capacity to clear the primary infection [44,45]. 

By applying ‘Birth and Death Skyline contemporary (BDSKYcontemp)’ and ‘Birth and Death Skyline serial (BDSKYserial)’ analyses for homochronous (SerD and non-SerD cohorts) and heterochronous (includes non-Italian references) sequences, respectively, the effective temporal Re could be estimated. When using homochronous sequences and applying a BDSKYcontemp model, there was no algorithm calibration based on sample dates, however, the analysis focused solely on query sequences. By including heterochronous sequences and applying a BDSKYserial model, there was greater confidence in the generated numbers, with the caveat that the results may have partially reflected population dynamics from the reference sequences. Both analyses indicated an overall constant population (Re ≈ 1), culminating in a very recent sharp increase. While this might be qualitatively true, Re numbers obtained using the BDSKYcontemp model were probably inflated, while Re calculated using the BDSKYserial model were conservative. Qualitatively, both analyses showed an increase in HCV infections over the past five years. 

The phylodynamic analysis was valuable in the identification of different clusters and transmission routes among PWUD, as with the participants infected with Gt2b [46,47]. These findings could not be extracted from traditional epidemiological analyses. The anti-HCV antibody-positivity for these Gt2b participants was established during different time intervals (between 1995 and 2019). Nevertheless, as stated in the questionnaire, the participants were friends who exchanged syringes while injecting heroin or cocaine. A limitation of the phylogenetic and phylodynamic analyses was the inability to identify whether a particular strain had a higher risk of transmission. Moreover, it is unknown whether other HCV-positive individuals registered at each SerD, but were not a part of our study, were infected with similar HCV strains and either recovered or progressed to chronic infection. Access to this type of information can only be obtained in a prospective study. Interestingly, none of our study sequences (beginning with either INMI, Pt, or Q) clustered with sequences from other countries. A possible explanation is the limited sample size. For example, no database HCV Gt3 sequences from Pakistan or Afghanistan, countries historically frequented by Italian PWUD, clustered with HCV NS5B sequences detected in our study participants. 

## 5. Conclusions

In this retrospective study, we demonstrated that despite national preventative measures against the spread of HCV infection, such as dissemination of information, needle exchange programs, and distribution of drug paraphernalia, HCV prevalence among PWUD is similar to that reported ten years ago [16]. Four of our HCV RNA-positive study participants declared only using drug paraphernalia, reinforcing how sniffing equipment represents an important factor in HCV transmission [8,48].

The National Surveillance System reported a gradual decrease in the incidence rate of HCV in Italy over the past twenty years [49]. In the last year, PWUD accounted for approximately 23.5% of these cases [49]. Antiviral treatments are still the best preventative intervention against disease progression and the spread of endemic HCV infection between PWUD since no HCV vaccines exist. DAAs as treatment options against all of the HCV genotypes have been crucial in curtailing the spread of the virus. Their use has led to a greater than 90% cure rate even among PWUD [50,51]. Therefore, a focus on treating PWUD communities with DAAs could be highly effective in controlling the transmission of HCV among this vulnerable population. 

## Figures and Tables

**Table 1:** Hepatitis C virus (HCV) genotype distribution in HCV RNA-positive people who use drugs (PWUD) and were recruited between 2018 and 2020.

Characteristics	Total (*n*)	HCV Genotype				
		1a	1b	2	3a	4
Age (years), (*n*) mean ± SD	(*n* = 101) 51 ± 8.4	(*n* = 45) 49 ± 9.0	(*n* = 4) 53 ± 7.1	(*n* = 4) 62 ± 5.5	(*n* = 36) 50 ± 8.5	(*n* = 12) 51 ± 3.2
HCV RNA (*n*)	(*n* = 101)	(*n* = 45)	(*n* = 4)	(*n* = 4)	(*n* = 36)	(*n* = 12)
viral load (IU/mL) mean	2,263,418	1,932,141	1,543,083	4,566,009	2,079,427	3,530,260
range	216–16,780,074	405–12,270,418	382,582–3,776,646	297,144–8,166,062	216–9,606,805	680–16,780,074

**Table 2:** Characteristics of SerD study participants with available HCV NS5B sequence and recruited between 2018 and 2019 (N.S. = not stated).

Characteristics	Total (*n*)	HCV Genotype				
		1a	1b	2	3a	4
Age (years), mean ± SD	50 ± 8.9 (*n* = 66)	49 ± 9.4 (*n* = 31)	57 ± 3.5 (*n* = 3)	61 ± 5.3 (*n* = 3)	48 ± 9.1 (*n* = 23)	52 ± 2.3 (*n* = 6)
≤34	5	3	-	-	2	-
35–44	7	4	-	-	3	-
≥45	54	24	3	3	18	6
Gender	(*n* = 66)	(*n* = 31)	(*n* = 3)	(*n* = 3)	(*n* = 23)	(*n* = 6)
Male	54	26	3	3	18	4
Female	12	5	-	-	5	2
Estimated year of HCV infection	(*n* = 66)	(*n* = 31)	(*n* = 3)	(*n* = 3)	(*n* = 23)	(*n* = 6)
<1990	7	5	-	-	1	1
1990–1995	22	8	1	1	9	3
1996–2001	13	6	1	1	4	1
2002–2007	7	1	1	-	4	1
2008–2013	10	7	-	-	3	-
2014–2019	6	3	-	1	2	-
NS	1	1	-	-	-	-
Assumption for infection	(*n* = 66)	(*n* = 31)	(*n* = 3)	(*n* = 3)	(*n* = 23)	(*n* = 6)
Inhalation	4	2	-	-	1	1
Intravenous	5	4	-	-	1	-
Inhal and Intrav	55	23	3	3	21	5
NS	2	2	-		-	-
Most commonly injected drug at recruitment	(*n* = 66)	(*n* = 31)	(*n* = 3)	(*n* = 3)	(*n* = 23)	(*n* = 6)
Heroin	20	9	-	2	8	1
Cocaine	1	1	-	-	-	-
Heroin and Cocaine	45	21	3	1	15	5

Abbreviations: Inhal, inhalation; Intra, intravenous; *n*, number; NS, not stated.

**Table 3:** Resulting clusters based on sequence similarity and posterior probability (>95%).

Id1	Id2	Genetic Similarity
INMI_109_LUCATT_gt1a_2019	INMI_173_STOGIA_gt1a_2019	0.026
INMI_11_MALLAU_gt1a_2019	INMI_23_MURMAU_gt1a_2019	0.030
INMI_114_FUBSAN_gt2_2019	INMI_120_GILENZ_gt2_2019	0.030
INMI_114_FUBSAN_gt2_2019	INMI_238_SGRENZ_gt2_2019	0.020
INMI_120_GILENZ_gt2_2019	INMI_238_SGRENZ_gt2_2019	0.030
INMI_136_DIGGAB_gt1_2019	Pt_201_MORMAR_1a_2017	0.020
INMI_140_SILARM_gt1a_2019	INMI_83_COMENR_gt1a_2019	0.026
INMI_140_SILARM_gt1a_2019	INMI_74_ASTALE_gt1a_2019	0.027
INMI_47_AMUASI_gt3_2019	INMI_48_FELEMI_gt3_2019	0.018

## Data Availability

The data presented in this study are available on request from the corresponding author.

## Supplementary Materials

The following are available online at https://www.mdpi.com/article/10.3390/microorganisms9071432/s1, Table S1: List of global GenBank hepatitis C virus (HCV) genome sequences used in phylogenetic and phylodynamic analyses; Table S2: non SerD patients included in our phylogenetic analysis; Table S3: SerD patients included in our phylogenetic analysis; Table S4: HCV genome reference sequences; Table S5: Estimation of temporal reproduction number distribution using a Birth-Death Skyline (contemporary) model for homochronous HCV sequences for (A) Gt1a and (B) Gt3a; Table S6: Estimation of temporal reproduction number distribution using a Birth-Death Skyline (serial) model for heterochronous HCV sequences (A) Gt1a and (B) Gt3a. “The study was conducted according to the guidelines of the Declaration of Helsinki, and approved by the Institutional Review Board of INMI L Spallanzani IRCCS (protocol code n 49/2017 and date of approval: 10 July 2017)”.
