# Supplementary material for: Phylogenetic and Phylodynamic Analyses of HCV Strains Circulating among Patients Using Injectable Drugs in Central Italy
Source: Microorganisms. 2021 Jul 2;9(7):1432. doi: 10.3390/microorganisms9071432 (PMC8304011; doi:10.3390/microorganisms9071432)
Supplement: Supplementary file 1 [file microorganisms-09-01432-s001.zip › Minosse et al_TableS1.pdf]

Table S1. List of global GenBank hepatitis C virus (HCV) genome sequences used in phylogenetic and phylodynamic analyses

GenBank sequences	Acc. Number	Genotype	Year	Country
1a.AU.2008.111.JX437205	JN437205	1a	2008	AU
1a.BR.2003.BR601.EF032886	EF032886	1a	2003	BR
1a.CH.2001.HCV-1a/CH/BID-V242/2001.EU482854	EU482854	1a	2001	CH
1a.CH.2002.HCV-1a/CH/BID-V249/2002.EU482855	EU482855	1a	2002	CH
1a.CH.2002.HCV-1a/CH/BID-V250/2002.EU482856	EU482856	1a	2002	CH
1a.CH.2003.HCV-1a/CH/BID-V256/2003.EU155350	EU155350	1a	2003	CH
1a.CH.2004.HCV-1a/CH/BID-V262/2004.EU155352	EU155352	1a	2004	CH
1a.CH.2005.HCV-1a/CH/BID-V240/2005.EU155348	EU155348	1a	2005	CH
1a.CH.2005.HCV-1a/CH/BID-V263/2005.EU482857	EU482857	1a	2005	CH
1a.CH.2006.HCV-1a/CH/BID-V271/2006.EU482858	EU482858	1a	2006	CH
1a.DE.2003.HCV-1a/DE/BID-V24/2003.EU862823	EU862823	1a	2003	DE
1a.DE.2003.HCV-1a/DE/BID-V25/2003.EU482831	EU482831	1a	2003	DE
1a.DE.2003.HCV-1a/DE/BID-V26/2003.EU155378	EU155378	1a	2003	DE
1a.DE.2003.HCV-1a/DE/BID-V27/2003.EU862832	EU862832	1a	2003	DE
1a.DE.2004.HCV-1a/DE/BID-V1563/2004.EU687194	EU687194	1a	2004	DE
1a.DE.2004.HCV-1a/DE/BID-V33/2004.EU482832	EU482832	1a	2004	DE
1a.DE.2004.HCV-1a/DE/BID-V35/2004.EU155380	EU155380	1a	2004	DE
1a.DE.2004.HCV-1a/DE/BID-V37/2004.EU862834	EU862834	1a	2004	DE
1a.DE.2005.HCV-1a/DE/BID-V1562/2005.EU687193	EU687193	1a	2005	DE
1a.DE.2007.HCV-1a/DE/BID-V1564/2007.EU687195	EU687195	1a	2007	DE
1a.NZ.2008.101.JX437195	JN437195	1a	2008	NZ
1a.NZ.2008.102.JX437196	JN437196	1a	2008	NZ
1a.NZ.2008.103.JX437197	JN437197	1a	2008	NZ
1a.NZ.2008.104.JX437198	JN437198	1a	2008	NZ
1a.NZ.2009.201.JX437212	JN437212	1a	2009	NZ
1a.NZ.2009.202.JX437213	JN437213	1a	2009	NZ
1a.NZ.2009.203.JX437214	JN437214	1a	2009	NZ
1a.US.1977.H77.AF009606	AF009606	1a	1977	US
1a.US.1990.HCV-1a/US/BID-V168/1990.EU255965	EU255965	1a	1990	US
1a.US.1990.HCV-1a/US/BID-V172/1990.EU155339	EU155339	1a	1990	US
1a.US.1991.HCV-1a/US/BID-V185/1991.EU155340	EU155340	1a	1991	US
1a.US.1992.HCV-1a/US/BID-V167/1992.EU255964	EU255964	1a	1992	US
1a.US.1992.HCV-1a/US/BID-V192/1992.EU155341	EU155341	1a	1992	US
1a.US.1996.HCV-1a/US/BID-V169/1996.EU155338	EU155338	1a	1996	US
1a.US.1997.HCV-1a/US/BID-V170/1997.EU255966	EU255966	1a	1997	US
1a.US.2000.HCV-1a/US/BID-V412/2000.EU155312	EU155312	1a	2000	US
1a.US.2001.HCV-1a/US/BID-V115/2001.EU155216	EU155216	1a	2001	US
1a.US.2001.HCV-1a/US/BID-V317/2001.EU155284	EU155284	1a	2001	US
1a.US.2002.HCV-1a/US/BID-V432/2002.EU155276	EU155276	1a	2002	US
1a.US.2002.HCV-1a/US/BID-V71/2002.EU155241	EU155241	1a	2002	US
1a.US.2003.HCV-1a/US/BID-V93/2003.EU155246	EU155246	1a	2003	US
1a.US.2004.HCV-1a/US/BID-V101/2004.EU155213	EU155213	1a	2004	US

1a.US.2004.HCV-1a/US/BID-V357/2004.EU155310	EU155310	1a	2004	US
1a.US.2005.HCV-1a/US/BID-V104/2005.EU155214	EU155214	1a	2005	US
1a.US.2005.HCV-1a/US/BID-V388/2005.EU155265	EU155265	1a	2005	US
1a.US.2006.HCV-1a/US/BID-V453/2006.EU255934	EU255934	1a	2006	US
1a.US.2006.HCV-1a/US/BID-V457/2006.EU155237	EU155237	1a	2006	US
1a.US.2007.HCV-1a/US/BID-V462/2007.EU255935	EU255935	1a	2007	US
1a.US.2007.HCV-1a/US/BID-V465/2007.EU155238	EU155238	1a	2007	US
1b.AU.2009.208.JX437219	JN437219	1b	2009	AU
1b.AU.2009.313.JX437234	JN437234	1b	2009	AU
1b.BR.2003.BR1427_P1_10-7-03.EF032892	EF032892	1b	2003	BR
1b.BR.2003.BR1427_P3_11-10-03.EF032893	EF032893	1b	2003	BR
1b.BR.2004.BR1427_P7_3-1-04.EF032894	EF032894	1b	2004	BR
1b.CH.2002.HCV-1b/CH/BID-V277/2002.EU155359	EU155359	1b	2002	CH
1b.CH.2002.HCV-1b/CH/BID-V291/2002.EU529682	EU529682	1b	2002	CH
1b.CH.2003.HCV-1b/CH/BID-V272/2003.EU482859	EU482859	1b	2003	CH
1b.CH.2003.HCV-1b/CH/BID-V275/2003.EU155357	EU155357	1b	2003	CH
1b.CH.2004.HCV-1b/CH/BID-V276/2004.EU155358	EU155358	1b	2004	CH
1b.CH.2004.HCV-1b/CH/BID-V304/2004.EU862837	EU862837	1b	2004	CH
1b.CH.2005.HCV-1b/CH/BID-V285/2005.EU155363	EU155363	1b	2005	CH
1b.CH.2005.HCV-1b/CH/BID-V287/2005.EU482874	EU482874	1b	2005	CH
1b.CH.2006.HCV-1b/CH/BID-V288/2006.EU155364	EU155364	1b	2006	CH
1b.CH.2006.HCV-1b/CH/BID-V312/2006.EU255960	EU255960	1b	2006	CH
1b.CN.2001.Whu.EU857431	EU857431	1b	2001	CN
1b.CN.2002.GZ51969.KC844052	KC844052	1b	2002	CN
1b.CN.2002.GZ52540.KC844051	KC844051	1b	2002	CN
1b.CN.2003.H1.GU451218	GU451218	1b	2003	CN
1b.CN.2003.S1.GU451221	GU451221	1b	2003	CN
1b.CN.2004.H3.GU451219	GU451219	1b	2004	CN
1b.CN.2004.S3.GU451223	GU451223	1b	2004	CN
1b.CN.2007.PR50.HQ912957	HQ912957	1b	2007	CN
1b.CN.2007.PR52.HQ912958	HQ912958	1b	2007	CN
1b.CN.2008.PR79.HQ912959	HQ912959	1b	2008	CN
1b.CN.2008.S4.GU451224	GU451224	1b	2008	CN
1b.DE.2003.HCV-1b/DE/BID-V503/2003.EU482833	EU482833	1b	2003	DE
1b.DE.2003.HCV-1b/DE/BID-V504/2003.EU155382	EU155382	1b	2003	DE
1b.DE.2004.HCV-1b/DE/BID-V502/2004.EU155381	EU155381	1b	2004	DE
1b.DK.2018.pt#7-Week0.MT995341	MT995341	1b	2018	DK
1b.DK.2018.pt#7-Week12.MT995342	MT995342	1b	2018	DK
1b.DK.2018.pt#7-Week20.MT995343	MT995343	1b	2018	DK
1b.DK.2018.pt#7-Week32.MT995344	MT995344	1b	2018	DK
1b.DK.2018.pt#7-Week40.MT995345	MT995345	1b	2018	DK
1b.FR.2003.Lex.JN120912	JN120912	1b	2003	FR
1b.JP.1988.blood_donor.D89815	D89815	1b	1988	JP
1b.JP.1991.AH1.AB429050	AB429050	1b	1991	JP
1b.JP.2008.HCV-K.AB249644	AB249644	1b	2008	JP

1b.NZ.2008.107.JX437201	JN437201	1b	2008	NZ
1b.NZ.2008.116.JX437210	JN437210	1b	2008	NZ
1b.NZ.2009.205.JX437216	JN437216	1b	2009	NZ
1b.NZ.2009.301.JX437222	JN437222	1b	2009	NZ
1b.RU.1999.N589.AY587844	AY587844	1b	1999	RU
1b.RU.2000.817-06.HQ641455	JN641455	1b	2000	RU
1b.RU.2000.895-06.HQ641456	JN641456	1b	2000	RU
1b.RU.2001.814-06.HQ641453	JN641453	1b	2001	RU
1b.RU.2002.176-04.GU254019	JN254019	1b	2002	RU
1b.RU.2002.187-04.GU254022	JN254022	1b	2002	RU
1b.RU.2006.820-06.HQ641454	JN641454	1b	2006	RU
1b.RU.2018.HCV_NWFD_1cg_coinf18.MT512570	MT512570	1b	2018	RU
1b.US.1989.HCV-1b/US/BID-V133/1989.EU155333	EU155333	1b	1989	US
1b.US.1990.HCV-1b/US/BID-V131/1990.EU155331	EU155331	1b	1990	US
1b.US.1991.HCV-1b/US/BID-V122/1991.EU155325	EU155325	1b	1991	US
1b.US.1992.HCV-1b/US/BID-V121/1992.EU155324	EU155324	1b	1992	US
1b.US.1994.HCV-1b/US/BID-V130/1994.EU155330	EU155330	1b	1994	US
1b.US.1995.C2.FJ380065	FJ380065	1b	1995	US
1b.US.1996.C1.FJ380064	FJ380064	1b	1996	US
1b.US.1997.C3.FJ380066	FJ380066	1b	1997	US
1b.US.1998.N5_post.FJ380078	FJ380078	1b	1998	US
1b.US.2001.HCV-1b/US/BID-V154/2001.EU660388	EU660388	1b	2001	US
1b.US.2002.HCV-1b/US/BID-V163/2002.EU155231	EU155231	1b	2002	US
1b.US.2003.HCV-1b/US/BID-V158/2003.EU155228	EU155228	1b	2003	US
1b.US.2003.HCV-1b/US/BID-V383/2003.EU482881	EU482881	1b	2003	US
1b.US.2004.HCV-1b/US/BID-V156/2004.EU155226	EU155226	1b	2004	US
1b.US.2005.HCV-1b/US/BID-V384/2005.EU155263	EU155263	1b	2005	US
1b.US.2006.HCV-1b/US/BID-V458/2006.EU482886	EU482886	1b	2006	US
1b.US.2007.HCV-1b/US/BID-V1715/2007.FJ024279	FJ024279	1b	2007	US
1b/2b.JP.2010.HC10-0804.AB622121	AB622121	1b	2010	JP
1b/2k.RU.1999.N687.AY587845	AY587845	1b	1999	RU
2.CM.2016.S6309.MH477423	JN477423	2	2016	CM
2.FR.1995.MRS40.KC197238	JN197238	2	1995	FR
2.FR.2001.MRS117.KC197237	JN197237	2	2001	FR
2.FR.2009.PTR9203.KC197239	JN197239	2	2009	FR
2a.CN.2011.ZS623.KC844043	KC844043	2a	2011	CN
2a.DK.2012.T9/JFH1.KC967476	KC967476	2a	2012	DK
2a.JP.2011.AB690460.AB690460	AB690460	2a	2011	JP
2a.US.2010.JFH1-AM120.KF700370	KF700370	2b	2010	US
2b.CN.2010.ZS260.KC844048	KC844048	2b	2010	CN
2b.DK.2012.J8CF.JQ745651	JQ745651	2b	2012	DK
2b.DK.2018.pt#6-Week32.MT995339	MT995339	2b	2018	DK
2b.DK.2018.pt#6-Week40.MT995340	MT995340	2b	2018	DK
2b.FR.2001.MRS129.KC197226	KC197226	2b	2001	FR
2b/1a.US.2014.Subject_24.MK548367	MK548367	2b	2014	US

2b/1a.US.2015.Subject_26.MK548369	MK548369	2b	2015	US
2c.DK.2012.S83/JFH1.KC967479	KC967479	2c	2012	DK
2c.FR.2004.FrBd3618.KC197228	KC197228	2c	2004	FR
2c.FR.2008.PTR1256.KC197227	KC197227	2c	2008	FR
2f.CN.2002.98799.KC844050	JN844050	2f	2002	CN
2f.CN.2010.ZS542.KC844042	KC844042	2f	2010	CN
2i.FR.1995.MRS37.KC197229	KC197229	2i	1995	FR
2i.FR.2001.MRS178.KC197231	KC197231	2i	2001	FR
2i.FR.2008.PTR9415.KC197230	KC197230	2i	2008	FR
2j.FR.2005.FrBd0565.KC197232	KC197232	2j	2005	FR
2j.FR.2009.PTR2795.KC197233	KC197233	2j	2009	FR
2j.VE.2005.C1292.HM777359	HM777359	2j	2005	VE
2k.FR.2009.PTR3874.KC197234	KC197234	2k	2009	FR
2k/1b.BE.2014.subject_23.MK527509	MK527509	2k/1b	2014	BE
2l.FR.1995.MRS89.KC197235	KC197235	2l	1995	FR
2l.FR.2008.PTR7904.KC197240	KC197240	2l	2008	FR
2q.ES.2002.963.FN666428	JN666428	2q	2002	ES
3.CA._.QC115.JF735124	JN735124	3	-	CA
3.CN.2016.2584.MN385583	MN385583	3	2016	CN
3a.CN.2008.PR87.HQ912953	HQ912953	3a	2008	CN
3a.CN.2011.ZS633.KC844041	KC844041	3a	2011	CN
3a.CN.2018.PR87A.MN231294	MN231294	3a	2018	CN
3a.DE._.HCVCENS1.X76918	X76918	3a	-	DE
3a.DK.2018.pt#8-Week32.MT995349	MT995349	3a	2018	DK
3a.DK.2018.pt#8-Week40.MT995350	MT995350	3a	2018	DK
3a.GB.2005.Patient_235.GQ356206	GQ356206	3a	2005	GB
3A.IL.2016.2000912.MT632158	JN632158	3a	2016	IL
3A.IL.2017.2001065.MT632172	JN632172	3a	2017	IL
3a.IN.2003.GQ275355.GQ275355	GQ275355	3a	2003	IN
3a.IN.2008.ILBSRAS9.JQ717260	JQ717260	3a	2008	IN
3a.IN.2010.ILBSRAS8.JQ717259	JQ717259	3a	2010	IN
3a.IN.2011.ILBSRAS7.JQ717258	JQ717258	3a	2011	IN
3a.IN.2012.ILBSRAS_UG.KF035127	KF035127	3a	2012	IN
3b.CN.2011.SH37.JQ065709	JQ065709	3b	2011	CN
3b.CN.2011.ZS650.KC844044	KC844044	3b	2011	CN
3i.IN.2002.IND-HCV-3i.FJ407092	FJ407092	3i	2002	IN
4.EG.2018.EG.MN782309	JN782309	4	2018	EG
4a.JP.2012.HCVgenotype4a-KM.AB795432	AB795432	4a	2012	JP
4d.CN.2010.ZS537.KC844045	KC844045	4d	2010	CN
4f.CM.2016.S6406.MH477424	MH477424	4f	2016	CM
4k.UG.2014.U275_Nakaseeta.MH742364	MH742364	4k	2014	UG
4k.UG.2014.U317_Butiti.MH742370	MH742370	4k	2014	UG
4q.UG.2014.U149_Bulwadda.MH742362	MH742362	4q	2014	UG
4q.UG.2014.U282_Nalunnya.MH742366	MH742366	4q	2014	UG
4s.UG.2014.U295_Busoga.MH742368	MH742368	4s	2014	UG

4v.UG.2014.U278_Nalunnya.MH742365	MH742365	4v	2014	UG
4v.UG.2014.U294_Busoga.MH742367	MH742367	4v	2014	UG
6.CN.2014.HCV074.MK327985	JN327985	6	2014	CN
6.CN.2014.HCV075.MK327996	JN327996	6	2014	CN
6.CN.2014.HCV076.MK328005	JN328005	6	2014	CN
6.CN.2014.HCV077.MK327984	JN327984	6	2014	CN
6.CN.2014.HCV092.MK328009	JN328009	6	2014	CN
6.CN.2014.HCV093.MK328001	JN328001	6	2014	CN
6.CN.2014.HCV094.MK328019	JN328019	6	2014	CN
6.CN.2014.HCV104.MK327998	JN327998	6	2014	CN
6.CN.2015.HCV084.MK328003	JN328003	6	2015	CN
6.CN.2015.HCV099.MK328006	JN328006	6	2015	CN
6.CN.2015.HCV101.MK327981	JN327981	6	2015	CN
6.CN.2015.HCV156.MK328013	JN328013	6	2015	CN
6.DK.2019.HVH-HCV334.MN240359	MN240359	6	2019	DK
6.MY.2010.10MYKJ032.KC191671	JN191671	6	2010	MY
6a.CN.2008.PR144.HQ912955	HQ912955	6a	2008	CN
6a.CN.2008.PR58.HQ912954	HQ912954	6a	2008	CN
6a.CN.2009.ZS221.KC844037	KC844037	6a	2009	CN
6a.CN.2011.ZS674.KC844038	KC844038	6a	2011	CN
6v.CN.2004.NK46.EU158186	EU158186	6v	2004	CN
