# Supplementary material for: Phylogenetic and Phylodynamic Analyses of HCV Strains Circulating among Patients Using Injectable Drugs in Central Italy
Source: Microorganisms. 2021 Jul 2;9(7):1432. doi: 10.3390/microorganisms9071432 (PMC8304011; doi:10.3390/microorganisms9071432)
Supplement: Supplementary file 1 [file microorganisms-09-01432-s001.zip › Minosse et al_TableS2.pdf]

Table S2. Non-SerD patients included in our phylogenetic analysis

Patients	Collection Date	Age (years)	Genotype	GenBank Acc.Number
Pt_156_MUSROB_2c_2016	05/11/2015	61	2c	MW927662
Pt_179_COCBRU_1a_2016	21/09/2016	56	1a	MW927663
Pt_191_MANGIU_4d_2016	02/11/2016	65	4d	MW927664
Pt_196_BERMAU_1a_2017	09/03/2017	54	1a	MW927665
Pt_197_MANMAR_3a_2017	05/04/2017	61	3a	MW927666
Pt_199_MATTAN_3a_2017	15/03/2017	41	3a	MW927667
Pt_201_MORMAR_1a_2017	30/03/2017	52	1a	MW927668
Pt_202_GATGIO_2c_2017	13/04/2017	72	2c	MW927669
Pt_203_CASSTE_1b_2017	11/04/2017	49	1b	MW927670
Pt_204_SERMAU_1b_2017	18/05/2017	81	1b	MW927671
Pt_208_MORMAU_1b_2017	30/05/2017	62	1b	MW927672
Pt_211_VILMAR_2c_2017	28/09/2017	77	2c	MW927673
Pt_213_BONPAO_2c_2017	30/09/2017	66	2c	MW927674
Pt_217_DANMAR_1a_2018	09/01/2018	51	1a	MW927675
Pt_221_ORTMIG_3a_2018	20/04/2018	44	3a	MW927676
Pt_222_DSCAGO_1b_2020	17/04/2020	80	1b	MW927677
Pt_223_UGUMIR_3a_2020	29/04/2020	51	3a	MW927678
Pt_224_GIUJUR_1a_2020	21/04/2020	29	1a	MW927679
Pt_225_LWICHO_6m_2019	27/07/2019	55	6m	MW927680
Pt_226_DIEGIN_2c_2019	24/08/2019	75	2c	MW927681
Pt_227_MARCHI_3a_2018	14/12/2018	54	3a	MW927682
Pt_228_UDDSHA_3b_2019	07/05/2019	41	3b	MW927683
Q0219_MARGIO_gt3a	17/01/2018	60	3a	MW927684
Q0482_BOCMAR_gt1b	02/02/2018	43	1b	MW927685
Q0531_SALCLA_gt1a	02/02/2017	58	1a	MW927686
Q0668_PETAGO_gt4d	10/02/2017	58	4d	MW927687
Q0870_ABBSAV_gt2c	24/02/2017	72	2c	MW927688
Q0889_GIRFRA_gt1a	25/02/2017	51	1a	MW927689
Q1134_SOLFAB_gt1b	11/03/2017	62	1b	MW927690
Q1282_CANLUI_gt3a	20/03/2017	54	3a	MW927691
Q1370_MARMAU_gt3a	03/05/2018	61	3a	MW927692
Q1603_PIEROM_gt2c	25/05/2018	79	2c	MW927693
Q1814_IACSAM_gt4d	10/06/2019	51	4d	MW927694
Q2030_CARRAF_gt2c	06/07/2019	76	2c	MW927695
Q2394_DIVANT_gt1a	18/11/2020	49	1a	MW927696
Q2811_AMOMAU_gt4d	01/10/2019	59	4d	MW927697
Q3383_ALFVIN_gt4d	07/07/2017	60	4d	MW927698
Q3514_POLMAR_gt2c	06/12/2019	79	2c	MW927699
Q3593_SAWMOO_gt6n	24/12/2016	42	6n	MW927700
Q3979_RAZROM_gt3a	16/08/2017	54	3a	MW927701
Q5594_GIOLUC_gt1b	19/10/2017	71	1b	MW927702
Q6164_IANMAU_gt1a	17/11/2017	45	1a	MW927703
