# Supplementary material for: Phylogenetic and Phylodynamic Analyses of HCV Strains Circulating among Patients Using Injectable Drugs in Central Italy
Source: Microorganisms. 2021 Jul 2;9(7):1432. doi: 10.3390/microorganisms9071432 (PMC8304011; doi:10.3390/microorganisms9071432)
Supplement: Supplementary file 1 [file microorganisms-09-01432-s001.zip › Minosse et al_TableS3.pdf]

Table S3. SerD patients included in our phylogenetic analysis

Patients	Collection Date	Age (years)	Genotype	GenBank Acc.Number
INMI_10_GIAANT	03/05/2018	57	3a	MW927598
INMI_107_DELGIO	04/07/2019	46	3a	MW927596
INMI_109_LUCATT	10/04/2019	62	1a	MW927597
INMI_11_MALLAU	03/05/2018	60	1a	MW927600
INMI_114_FUBSAN	13/04/2019	55	2	MW927599
INMI_120_GILENZ	21/03/2019	63	2	MW927601
INMI_13_CELSIL	12/11/2018	37	1a	MW927605
INMI_132_CAPALB	04/06/2019	55	1a	MW927602
INMI_133_CAREMA	13/04/2019	51	3a	MW927603
INMI_136_DIGGAB	20/03/2019	48	1a	MW927604
INMI_140_SILARM	29/04/2019	55	1a	MW927606
INMI_148_MEZSIM	11/03/2019	33	1a	MW927607
INMI_152_BARMAS	30/04/2019	66	3a	MW927608
INMI_153_BRAPIE	21/01/2020	51	1a	MW927609
INMI_154_CAPALF	22/05/2019	51	4	MW927610
INMI_159_PAGLUC	12/06/2019	53	1a	MW927611
INMI_162_URBALE	17/04/2019	49	3a	MW927612
INMI_170_MONFAB	10/04/2019	56	1a	MW927613
INMI_173_STOGIA	15/05/2019	54	1a	MW927614
INMI_178_BINAND	13/05/2019	59	3a	MW927615
INMI_180_GENFED	04/06/2019	40	3a	MW927616
INMI_182_PASGER	15/07/2019	47	3a	MW927617
INMI_188_SABBRU	02/04/2019	30	1a	MW927618
INMI_189_SANGIO	11/04/2019	40	1a	MW927619
INMI_192_TRIDAV	13/05/2019	52	1a	MW927620
INMI_2_BONUMB	05/04/2018	50	1a	MW927636
INMI_207_OTTGIA	08/07/2019	46	1a	MW927621
INMI_212_CIPRIT	03/07/2019	51	4	MW927622
INMI_213_DEPMAR	07/05/2019	56	4	MW927623
INMI_223_DISGIO	31/05/2019	54	3a	MW927624
INMI_226_BARVIR	30/07/2019	53	1b	MW927625
INMI_23_MURMAU	26/06/2018	60	1a	MW927631
INMI_231_BUSGIA	15/07/2019	45	3a	MW927626
INMI_232_CARMAR	12/06/2019	53	3a	MW927627
INMI_234_MARSTE	12/06/2019	56	1a	MW927628
INMI_237_DECFAB	03/08/2019	53	3a	MW927629
INMI_238_SGRENZ	12/06/2019	65	2	MW927630
INMI_255_CUGROB	22/05/2019	50	4	MW927632
INMI_261_FALBRU	30/05/2019	60	1b	MW927633
INMI_262_MIOFAB	15/07/2019	50	1a	MW927634
INMI_266_ANGSER	12/06/2019	54	3a	MW927635

INMI_31_GALMAS	25/08/2018	45	1a	MW927638
INMI_313_LENMAR	06/11/2019	47	3a	MW927637
INMI_322_PICROC	24/09/2019	60	3a	MW927639
INMI_337_LIGFRA	18/09/2019	50	1a	MW927640
INMI_35_VENMAR	29/08/2018	45	1a	MW927642
INMI_357_CASMAS	14/10/2019	51	4o	MW927641
INMI_36_BREALE	31/10/2018	42	3a	MW927644
INMI_365_BARCAR	06/11/2019	54	3a	MW927643
INMI_37_SINLUC	27/08/2018	45	3a	MW927645
INMI_38_CECSTE	04/06/2019	67	1a	MW927647
INMI_386_GESLAU	15/01/2020	45	3a	MW927646
INMI_395_PICPAO	04/12/2019	54	4	MW927648
INMI_411_DEVGIU	05/12/2019	44	1a	MW927649
INMI_419_ADAALE	10/12/2019	53	1a	MW927650
INMI_420_BRURIC	15/01/2020	41	3a	MW927651
INMI_47_AMUASI	31/10/2018	24	3a	MW927652
INMI_48_FELEMI	06/02/2019	33	3a	MW927653
INMI_49_VENRENGIU	08/11/2018	57	1b	MW927654
INMI_52_CARIDO	07/11/2018	47	1a	MW927655
INMI_55_FINALI	23/01/2019	27	1a	MW927656
INMI_61_DERALE	29/12/2018	52	1a	MW927657
INMI_74_ASTALE	27/02/2019	36	1a	MW927658
INMI_83_COMENR	15/05/2019	55	1a	MW927659
INMI_88_SCACAR	29/04/2019	50	3a	MW927660
INMI_90_DIGEMI	20/02/2019	55	1a	MW927661
