# Supplementary material for: Phylogenetic and Phylodynamic Analyses of HCV Strains Circulating among Patients Using Injectable Drugs in Central Italy
Source: Microorganisms. 2021 Jul 2;9(7):1432. doi: 10.3390/microorganisms9071432 (PMC8304011; doi:10.3390/microorganisms9071432)
Supplement: Supplementary file 1 [file microorganisms-09-01432-s001.zip › Minosse et al_TableS4.pdf]

Table S4. HCV genome reference sequences

Reference	GenBank Acc.Number	Genotype
AB661382_gt2b	AB661382	2b
AB661388_gt2b	AB661388	2b
D00944_gt2a	D00944	2a
D50409_gt2c	D50409	2c
DQ278894_gt6n	DQ278894	6n
DQ418786_gt4d	DQ418786	4d
DQ418789_gt4a	DQ418789	4a
DQ835766_gt6m	DQ835766	6m
DQ835767_gt6m	DQ835767	6m
DQ988074_gt4a	DQ988074	4a
EF108306_gt7a	EF108306	7a
EF407457_gt1a	EF407457	1a
EU246938_gt6n	EU246938	6n
EU392172_gt4d	EU392172	4d
EU781827_gt1b	EU781827	1b
EU781828_gt1b	EU781828	1b
FJ462440_gt4o	FJ462440	4o
HQ639944_gt2a	HQ639944	2a
HQ850279_gt1a	HQ850279	1a
X76918_gt3a	X76918	3a
JN714194_gt3a	JN714194	3a
JX227949_gt2c	JX227949	2c
JX227977_gt4o	JX227977	4o
