# Supplementary material for: Phylogenetic and Phylodynamic Analyses of HCV Strains Circulating among Patients Using Injectable Drugs in Central Italy
Source: Microorganisms. 2021 Jul 2;9(7):1432. doi: 10.3390/microorganisms9071432 (PMC8304011; doi:10.3390/microorganisms9071432)
Supplement: Supplementary file 1 [file microorganisms-09-01432-s001.zip › Minosse et al_TableS5.pdf]

Table S5. Estimation of temporal reproduction number distribution using a Birth-Death Skyline (contemporary) model for homochronous HCV sequences for A) Gt1a and B) Gt3a. For these analyses, a GTR gamma site model (4 categories) was selected with a strict molecular clock with sequence date constraints where possible.

A)	Gt1a - Birth and Death Skyline Contemporary (homochronous sequences)									
**Summary Statistic**	**Rep.Num. cont1**	**Rep.Num. cont2**	**Rep.Num. cont3**	**Rep.Num. cont4**	**Rep.Num. cont5**	**Rep.Num. cont6**	**Rep.Num. cont7**	**Rep.Num. cont8**	**Rep.Num. cont9**	**Rep.Num. Cont10**
**mean**	1.1699	1.1043	1.0711	1.0577	0.982	0.98	0.9266	0.9377	2.7836	25.0669
**stderr of mean**	0.0172	0.017	0.0159	0.0163	0.0148	0.0139	0.0141	0.0235	0.1566	0.8245
**stdev**	1.1087	1.0654	1.0026	1.0494	0.9284	0.9181	0.8606	0.9191	2.9815	20.8542
**variance**	1.2292	1.135	1.0052	1.1013	0.862	0.8429	0.7407	0.8447	8.8891	434.8959
**median**	0.8297	0.7992	0.7747	0.7532	0.7074	0.7119	0.6712	0.6674	1.7792	19.002
**value range**	[0.0283 - 21.1446]	[0.0268 - 22.7161]	[0.0227 - 13.3908]	[0.0179 - 15.5968]	[0.023 - 12.9868]	[0.0195 - 9.6884]	[0.0186 - 10.5462]	[0.0304 - 11.8969]	[0.012 - 37.6422]	[2.85 - 308.726]
**geometric mean**	0.8057	0.7741	0.7426	0.7372	0.689	0.6871	0.655	0.6534	1.594	19.5349
**95% HPD interval**	[0.0412 - 3.3206]	[0.0268 - 2.9997]	[0.0227 - 2.9494]	[0.0532 - 2.9422]	[0.0255 - 2.7164]	[0.0316 - 2.7282]	[0.0286 - 2.5637]	[0.0348 - 2.615]	[0.0347 - 8.6137]	[2.85 - 64.1684]
**auto-correlation time (ACT)**	2155.9679	2285.2482	2273.5997	2171.0787	2299.0922	2058.4751	2419.0874	5896.7084	24831.4393	14070.2402
**effective sample size (ESS)**	4174.9	3938.7	3958.9	4145.9	3915	4372.7	3720.8	1526.4	362.5	639.7

B)	Gt3a - Birth and Death Skyline Contemporary (homochronous sequences)									
**Summary Statistic**	**Rep.Num. cont1**	**Rep.Num. cont2**	**Rep.Num. cont3**	**Rep.Num. cont4**	**Rep.Num. cont5**	**Rep.Num. cont6**	**Rep.Num. cont7**	**Rep.Num. cont8**	**Rep.Num. cont9**	**Rep.Num. Cont10**
**mean**	1.179	1.1372	1.0932	1.0833	1.2176	1.0682	1.1117	1.8284	7.3014	18.7462
**stderr of mean**	0.0116	0.0113	0.0129	0.0438	0.21	0.0861	0.1301	0.4421	2.4613	3.0003
**stdev**	1.0982	1.0752	1.0428	1.1513	2.5369	1.453	1.8853	4.5969	13.2584	20.1342
**variance**	1.2061	1.156	1.0874	1.3256	6.4358	2.1113	3.5545	21.1315	175.7852	405.3873
**median**	0.8393	0.8094	0.7807	0.7526	0.74	0.7187	0.7087	0.7336	1.2101	12.6376
**value range**	[0.0203 - 14.0936]	[0.016 - 17.6834]	[0.0275 - 13.244]	[0.0171 - 22.5855]	[0.0291 - 142.0836]	[0.0141 - 34.379]	[0.0124 - 52.6456]	[0.0351 - 91.78]	[0.0231 - 202.5518]	[0.0575 - 241.8379]
**geometric mean**	0.811	0.7902	0.7617	0.7347	0.7327	0.7085	0.6989	0.7945	1.8925	10.3829

**95% HPD interval**	[0.0496 - 3.3426]	[0.0334 - 3.1941]	[0.0275 - 3.0305]	[0.0363 - 3.0696]	[0.0311 - 3.1558]	[0.0348 - 2.9211]	[0.0348 - 3.0609]	[0.0351 - 6.6435]	[0.0231 - 33.1138]	[0.0575 - 56.6407]
**auto-correlation time (ACT)**	10000	10000	13768.8667	1.30E+05	6.17E+05	3.16E+05	4.29E+05	8.32E+05	3.10E+06	2.00E+06
**effective sample size (ESS)**	9001	9001	6537.2	692	145.9	285	209.8	108.1	29	45
