# Supplementary material for: Phylogenetic and Phylodynamic Analyses of HCV Strains Circulating among Patients Using Injectable Drugs in Central Italy
Source: Microorganisms. 2021 Jul 2;9(7):1432. doi: 10.3390/microorganisms9071432 (PMC8304011; doi:10.3390/microorganisms9071432)
Supplement: Supplementary file 1 [file microorganisms-09-01432-s001.zip › Minosse et al_TableS6.pdf]

Table S6. Estimation of temporal reproduction number distribution using a Birth-Death Skyline (serial) model for heterochronous HCV sequences for A) Gt1a and B) Gt3a. For these analyses, a GTR gamma site model (4 categories) was selected with a strict molecular clock with sequence date constraints where possible.

**A)**	**Gt1a - Birth and Death Skyline Serial (with heterochronous reference sequences)**									
**Summary Statistic**	**Rep.Num. ser1**	**Rep.Num. ser2**	**Rep.Num. ser3**	**Rep.Num. ser4**	**Rep.Num. ser5**	**Rep.Num. ser6**	**Rep.Num. ser7**	**Rep.Num. ser8**	**Rep.Num. ser9**	**Rep.Num. ser10**
mean	1.5479	1.283	1.4067	1.5229	0.9763	1.8373	0.8858	1.8074	0.2154	2.118
stderr of mean	0.0105	7.61E-03	0.0113	0.0168	0.023	0.0182	7.27E-03	5.64E-03	2.04E-03	4.15E-03
stdev	0.6206	0.4382	0.3665	0.4275	0.4838	0.4343	0.2673	0.2351	0.1078	0.194
variance	0.3852	0.192	0.1343	0.1827	0.2341	0.1886	0.0715	0.0553	0.0116	0.0376
median	1.5431	1.2729	1.3805	1.5298	0.9218	1.8346	0.8696	1.7853	0.1998	2.1161
value range	[0.0482 - 5.2719]	[0.0541 - 3.7925]	[0.2351 - 3.6463]	[0.0834 - 3.3931]	[0.036 - 4.0929]	[0.1529 - 4.2651]	[0.0488 - 2.4373]	[1.15 - 3.3258]	[0.0124 - 0.6533]	[1.4734 - 3.2902]
geometric mean	1.3904	1.1946	1.3571	1.4524	0.8467	1.7806	0.8426	1.7927	0.1876	2.1092
95% HPD interval	[0.2342 - 2.6779]	[0.4141 - 2.1706]	[0.7311 - 2.1739]	[0.7018 - 2.3839]	[0.1271 - 1.8762]	[0.9718 - 2.6893]	[0.3656 - 1.4315]	[1.3798 - 2.2674]	[0.0351 - 0.4265]	[1.7265 - 2.4958]
auto-correlation time (ACT)	25888.7019	27119.8096	85455.2471	1.39E+05	2.04E+05	1.58E+05	66497.517	51850.4486	32404.581	41148.3108
effective sample size (ESS)	3476.8	3319	1053.3	649.8	441.5	571.2	1353.6	1736	2777.7	2187.5

**B)**	**Gt3a - Birth and Death Skyline Serial (with heterochronous reference sequences)**									
**Summary Statistic**	**Rep.Num. ser1**	**Rep.Num. ser2**	**Rep.Num. ser3**	**Rep.Num. ser4**	**Rep.Num. ser5**	**Rep.Num. ser6**	**Rep.Num. ser7**	**Rep.Num. ser8**	**Rep.Num. ser9**	**Rep.Num. ser10**
mean	1.6017	1.2753	1.131	1.5586	2.2974	2.1265	1.2903	1.6209	0.7808	4.0375
stderr of mean	0.0189	0.0129	0.0157	0.0258	0.03	0.0254	0.0172	0.012	6.51E-03	0.0225
stdev	1.1468	0.947	0.8825	1.1228	1.2448	1.0704	0.7614	0.676	0.4409	1.1653
variance	1.3151	0.8968	0.7788	1.2607	1.5495	1.1457	0.5798	0.457	0.1944	1.3579
median	1.3537	1.0467	0.8911	1.3303	2.1885	2.0168	1.1846	1.5572	0.7134	3.8584
value range	[0.0275 - 9.4154]	[0.0185 - 9.8026]	[0.0243 - 7.7233]	[0.0182 - 9.6665]	[0.0499 - 12.365]	[0.0316 - 8.3218]	[0.0275 - 7.2939]	[0.068 - 6.8398]	[0.0308 - 3.7874]	[1.5426 - 10.9997]
geometric mean	1.1831	0.9485	0.8324	1.1588	1.9082	1.8319	1.0569	1.4741	0.6554	3.8835
95% HPD interval	[0.058 - 3.7762]	[0.0504 - 3.1188]	[0.0243 - 2.8636]	[0.0475 - 3.7554]	[0.1054 - 4.4933]	[0.1919 - 4.0915]	[0.0765 - 2.7055]	[0.3255 - 2.8535]	[0.0687 - 1.6098]	[2.0762 - 6.3402]

auto-correlation time (ACT)	24483.1004	16807.0898	28550.7115	47562.4249	52281.9587	50628.9133	46169.7142	28258.5842	19620.4856	33577.8801
effective sample size (ESS)	3676.4	5355.5	3152.6	1892.5	1721.6	1777.8	1949.5	3185.2	4587.6	2680.6
